# Effects of ocean acidification on growth and photophysiology of two tropical reef macroalgae

**DOI:** 10.1371/journal.pone.0286661

**Published:** 2023-11-17

**Authors:** Heather N. Page, Sophie McCoy, Robert G. M. Spencer, Katherine A. Burnham, Clay Hewett, Maggie Johnson

**Affiliations:** 1 Elizabeth Moore International Center for Coral Reef Research and Restoration, Mote Marine Laboratory, Summerland Key, FL, United States of America; 2 Sea Education Association, Woods Hole, MA, United States of America; 3 University of North Carolina at Chapel Hill, Chapel Hill, NC, United States of America; 4 Florida State University, Tallahassee, FL, United States of America; 5 Jacksonville University, Jacksonville, Fl, United States of America; 6 Smithsonian Marine Station, Fort Pierce, FL, United States of America; The University of Auckland - City Campus: University of Auckland, NEW ZEALAND

## Abstract

Macroalgae can modify coral reef community structure and ecosystem function through a variety of mechanisms, including mediation of biogeochemistry through photosynthesis and the associated production of dissolved organic carbon (DOC). Ocean acidification has the potential to fuel macroalgal growth and photosynthesis and alter DOC production, but responses across taxa and regions are widely varied and difficult to predict. Focusing on algal taxa from two different functional groups on Caribbean coral reefs, we exposed fleshy (*Dictyota* spp.) and calcifying (*Halimeda tuna*) macroalgae to ambient and low seawater pH for 25 days in an outdoor experimental system in the Florida Keys. We quantified algal growth, calcification, photophysiology, and DOC production across pH treatments. We observed no significant differences in the growth or photophysiology of either species between treatments, except for lower chlorophyll *b* concentrations in *Dictyota* spp. in response to low pH. We were unable to quantify changes in DOC production. The tolerance of *Dictyota* and *Halimeda* to near-future seawater carbonate chemistry and stability of photophysiology, suggests that acidification alone is unlikely to change biogeochemical processes associated with algal photosynthesis in these species. Additional research is needed to fully understand how taxa from these functional groups sourced from a wide range of environmental conditions regulate photosynthesis (via carbon uptake strategies) and how this impacts their DOC production. Understanding these species-specific responses to future acidification will allow us to more accurately model and predict the indirect impacts of macroalgae on coral health and reef ecosystem processes.

## Introduction

Benthic macroalgae play important roles in biogeochemical and ecological processes on coral reefs, exerting their influence on larger scale community structure and ecosystem function. By utilizing the dissolved inorganic carbon (DIC) available in seawater for photosynthesis, benthic macroalgae elevate seawater pH [1,2] and oxygen [3] during the day. Most of this carbon is fixed, or assimilated into biomass, and subsequently grazed by herbivores, decomposed by bacteria, and/or buried in sediments [4]. However, up to 50% of this fixed carbon, in the form of dissolved organic carbon (DOC), can ‘leak’ from algal cells into the water column as algal exudates [5–8]. The release of DOC by macroalgae can modify the outcome of coral-algal competition [9–12], shift coral microbial communities to favor pathogens [12–15], and fuel sponge [16–19] and microbial [7,8,20–22] loops, all of which which decrease coral reef resilience to environmental stressors [23].

The influence of macroalgae on carbon cycling on tropical coral reefs may change in response to global climate change. The absorption of rapidly increasing atmospheric CO_2_ by global ocean surface waters [24] has led to a shift in seawater carbonate chemistry, increasing *p*CO_2_ (partial pressure of carbon dioxide), lowering carbonate ion concentrations [CO_3_^2-^], and decreasing the pH in a process known as ocean acidification [25]. Ocean acidification has been documented globally in open ocean surface waters since the 1980s [26,27], and seawater pH is now projected to decrease by an additional 0.05–0.4 units by the year 2100 under a range of trajectories for atmospheric greenhouse gas concentrations [28]. Nearshore, shallow marine habitats like coral reefs may experience even more extreme changes in seawater carbonate chemistry and pH in response to coastal processes (i.e., coastal acidification) [29].

Ocean and coastal acidification have been shown to impact the growth and photo-physiology of reef macroalgae. Many studies demonstrate no change or increased growth rates and photosynthesis in non-calcifying (i.e., fleshy) macroalgae under elevated seawater *p*CO_2_ [30–32]. This effect is of particular concern as fleshy macroalgae have stronger competitive strength against corals [33,34] than calcifying algae. In contrast, calcifying algae have generally been thought to respond negatively to ocean acidification due to lower seawater pH and Ω_CaCO3_ (saturation state with respect to calcium carbonate) that both facilitate CaCO_3_ dissolution and make it more difficult to build skeletal structures. Growth and/or calcification rates in these algae can be lower under acidification scenarios [31,35], especially at night (i.e., dark calcification) [36]. However, in a quantitative synthesis, Page et al. [37] found that almost half the studies on calcifying algae responses to ocean acidification report no change in calcification rates in laboratory experiments or field studies [35]. In other words, most calcifying algae maintain net photosynthesis and respiration rates when exposed to elevated seawater *p*CO_2_ and lower pH [35–37]. Inconsistencies in the physiological response of various macroalgae species to acidification pose uncertainties for how coral reefs will respond to global climate change.

Impacts on algal growth and photo-physiology under acidification could easily scale up to influence coral reef community structure and ecosystem function [38], both of which have already undergone significant changes in recent years. In the Caribbean, coral cover has declined [39–44] in the last few/couple of decades in response to local, regional, and global stressors, and these reefs are now being described as macroalgal dominated [41]. In addition to an increase in the abundance of benthic algae, the decline in coral cover on some reefs has been accompanied by greater presence of cyanobacterial mats and sponges [41,43,44]. Coral reef community structure, ecosystem processes and function, and reef resilience are therefore all impacted by macroalgal growth. Understanding the response of specific algal species and functional groups (i.e., non-calcifying and calcifying macroalgae) in the Caribbean is crucial for making informed predictions about the health of these reefs under global climate change. In this study, we investigated growth and photophysiology responses of non-calcifying (*Dictyota* spp., hereafter *Dictyota*) and calcifying (*Halimeda tuna*, hereafter *Halimeda*) macroalgae to short-term exposure (25 days) to elevated seawater *p*CO_2_ using an onshore ocean acidification simulator system in the Florida Keys.

## Materials and methods

### Specimen collection

Macroalgae were collected from rocky and coral reef environments nearshore to Summerland Key, Florida, USA. No permits are needed for collection of algae in the Florida Keys National Marine Sanctuary. Specimens were transported to Mote Marine Laboratory’s Elizabeth Moore International Center for Coral Reef Research and Restoration (Summerland Key, FL), where they were cleaned of epiphytes and sediments before being placed in shaded, flow-through aquaria with filtered ambient seawater maintained at 27°C. Macroalgae were acclimated to these conditions for one week before initial data collection and the start of the experiment.

### Experimental system and design: Climate and acidification ocean simulator

The acclimation and experiment were conducted in an outdoor experimental facility designed for climate change and ocean acidification studies. The Climate and Acidification Ocean Simulator (CAOS) contains six 5,000 L header (i.e., mixing) tanks and twelve 1,000 L raceways where experimental tanks are housed. Seawater was pumped from the Atlantic side of the Florida Keys through sand and 20 μm particle filters into a storage tank, where it could ‘off-gas’ excess CO_2_ to more closely mimic open ocean conditions (i.e., pH 8.0). The seawater was then pumped into two header tanks. The temperature and pH of each header tank were independently controlled and monitored using Walchem W900 controllers (Iwaki America Inc., Holliston, MA). Both header tanks were maintained at the same temperature (28.5°C). One tank contained seawater at ambient pH (~8.0) while the other was acidified to a target pH of 7.75 (ΔpH = 0.25). This pH offset represents an ocean acidification projection for the year 2100 under a moderate CO_2_ atmospheric concentration scenario (SSP2-4.5) [45]. Acidification was achieved by injecting CO_2_ into the header tank using a Venturi pump. Header tanks were rotated every two weeks to minimize any effects of biofouling on water quality. Seawater pH was gradually decreased to target levels over four days before the experiment began.

Seawater was delivered from the header tanks to 9.5 L flow-through experimental tanks using aquarium manifolds. Water flow into the experimental tanks was maintained daily at 180 mL min^-1^. These tanks were housed within two shallow raceways that served as a temperature-controlled water bath, which was maintained using a dual heat exchange system. The raceways were kept under a mesh shade cover to modulate light intensity. Mean (±1 sd) light within the raceways at 1330 EST (i.e., solar noon) was 377 ± 183 μE m^-2^ s^-1^. Each raceway contained twenty experimental tanks that received either ambient (pH 8.0) or acidified (pH 7.7) seawater (n = 40 tanks total). Tanks were cleaned bi-weekly to remove diatoms and other organisms that naturally recruited to and grew inside the tanks. In a balanced experimental design, each tank (n = 20 per pH treatment) contained one replicate *Dictyota* clump or *Halimeda* thallus that was tied to a small piece of pre-leached plastic eggcrate to keep the macroalgae submerged and in the proper orientation. Macroalgae were weighed at the start of the experiment to ensure similar biomass across all replicates (*Dictyota*: 1.905 ± 0.038 g; *Halimeda*: 0.924 ± 0.403 g). All replicates started the 25-day experiment healthy and with good coloration.

### Water chemistry monitoring

Throughout the experiment, the temperature and pH of the header tanks and subset of experimental tanks (n = 8 out of 40) were continuously monitored using Walchem monitoring systems. The probes were cleaned and calibrated weekly. The pH probes were calibrated against National Bureau of Standards (NBS) buffers and replaced every other week to ensure good performance. Seawater temperature (±0.2°C), salinity (±1.0% of the reading), dissolved oxygen, and pH_NBS_ of all tanks were measured daily at 0900 EST using a handheld YSI Professional Plus handheld multi-parameter instrument (Xylem Inc.). Salinity was calibrated weekly while dissolved oxygen and pH_NBS_ were calibrated daily. Water samples were collected from each header tank (n = 2) and a random subset of experimental tanks (n = 20; five per species × pH treatment) every three days for carbonate chemistry measurements. Best practices for seawater CO_2_ measurements [46] were followed during sampling, preservation, and laboratory analyses.

Dissolved inorganic carbon was measured using an Apollo SciTech AS-C3 DIC Analyzer (Apollo SciTech, Newark, DE) equipped with a LI-COR LI-7000 gas analyzer (LI-COR Biosciences, Lincoln, NE). Total alkalinity titrations were performed using a Metrohm 905 Titrando titrator (Metrohm, Herisau, Switzerland). Accuracy and precision were regularly checked using Certified Reference Materials for Seawater CO_2_ Measurements (Dickson Laboratory, Scripps Institution of Oceanography, UCSD, San Diego, CA). Accuracy and precision were less than 2 μmol kg^-1^ for dissolved inorganic carbon and less than 4 μmol kg^-1^ for total alkalinity. Seawater temperature, salinity, dissolved inorganic carbon, and total alkalinity were used to calculate pH_T_, *p*CO_2_, CO_2_, HCO_3_^-^, CO_3_^2-^, and Ω_arag_ using the software program CO2SYS [47]. K1 and K2 constants from Mehrbach et al. (1973) refit by Dickson and Millero (1987) were used in these calculations [48,49]. The seawater conditions (mean ± 1 sd) measured in the experimental tanks are presented in Table 1.

**Table 1:** Seawater chemistry in the experimental tanks.

	*Dictyota*		*Halimeda*	
pH Treatment:	Ambient	Low	Ambient	Low
**T (°C)**	27.2 ± 0.3	27.2 ± 0.4	27.2 ± 0.3	27.2 ± 0.3
**Salinity**	38.06 ± 0.29	38.08 ± 0.25	38.08 ± 0.28	38.08 ± 0.26
**DO (mg/L)**	6.18 ± 0.31	6.02 ± 0.24	6.22 ± 0.30	6.01 ± 0.26
**DIC (μmol/kg)**	2061 ± 39	2198 ± 51	2056 ± 38	2205 ± 49
**TA (μmol/kg)**	2358 ± 44	2355 ± 41	2355 ± 43	2356 ± 42
**pH**_**T**_	7.96 ± 0.02	7.69 ± 0.06	7.92 ± 0.01	7.67 ± 0.05
***p*CO**_**2**_ **(μatm)**	502 ± 29	1055 ± 152	546 ± 12	1098 ± 144
**CO**_**2**_ **(μmol/kg)**	13.2 ± 0.8	27.8 ± 3.9	14.3 ± 0.2	28.9 ± 3.7
**HCO**_**3**_^**-**^ **(μmol/kg)**	1835 ± 37	2044 ± 57	1823 ± 4	2054 ± 53
**CO**_**3**_^**2-**^ **(μmol/kg)**	212 ± 10	127 ± 14	193 ± 3	123 ± 13
**Ω**_**arag**_	3.33 ± 0.16	1.98 ± 0.21	3.02 ± 0.03	1.92 ± 0.20

Temperature (T), Salinity, Dissolved Oxygen (DO), DIC, and TA were directly measured while the other parameters were calculated using CO2SYS.

### Macroalgal photophysiological responses to ocean acidification

At the beginning and end of the experiment, we measured the wet weight of *Dictyota* replicates, after blotting excess water (blotted mass), to calculate growth. We measured the buoyant weight of *Halimeda* replicates at the beginning and end of the experiment to estimate growth as net calcification [50,51]. Buoyant weight was converted to actual weight by correcting for seawater density and density of aragonite (2.93 g cm^-3^), which is the polymorph of CaCO_3_ produced *Halimeda* [52]. Each *Halimeda* replicate was photographed and analyzed for surface area using ImageJ image processing software [53].

Net photosynthesis and respiration were measured by incubating macroalgal replicates in 300 mL respirometry chambers equipped with FireSting O_2_ optical oxygen sensors (PyroScience GmbH, Aachen, Germany) for 30 minutes. Control chambers (i.e., no algae) were included during each round of incubations to control for background oxygen fluxes in the water column (e.g., from microbial activity). The respirometry chambers were filled with fresh seawater from the same treatment as the algae’s experimental tank. Temperature was maintained at 27°C throughout the incubation using an external water bath. Magnetic stir bars isolated at the bottom of the chambers ensured mixing of seawater. Photosynthesis was measured under a mean light saturation of 122 ± 45 μE m^-2^ s^-1^ while respiration was measured in a dark room. Rates were calculated from the measured slope of oxygen change over time. For *Halimeda* and control incubations, total alkalinity was sampled at the beginning and end of the incubation to calculate light and dark calcification rates. Samples were analyzed immediately using the same procedures outlined for water chemistry monitoring. Net calcification rates were calculated from the change in total alkalinity over time. All physiological rates were standardized to algal biomass.

Maximum quantum yield provides an estimate of the photosynthetic efficiency of photosystem II (PSII) and serves as a proxy for algal photosynthetic performance. We measured the maximum quantum yield (Fv/Fm) of algae after exposure to treatment conditions using a red Pulse Amplitude Modulation Fluorometer (Diving PAM, Heinz Walz GmbH, Effeltrich, Germany). Individuals were dark adapted for at least one hour, and three measurements were taken from each individual in total darkness. For *Dictyota* the probe was placed haphazardly in three locations on the algal sample. For *Halimeda* the probe was placed ~1 cm from the growing tip (on the second youngest segment) of three separate segments on an individual. We calculated an average of the three points to account for variation in fluorescence-based measurements within an individual and used this average in subsequent analyses. Measurement settings were adjusted for optimal instrument performance as the following: saturation intensity = 8, saturating pulse width = 0.8, frequency = 2, gain = 2, measuring light intensity = 9. Samples were frozen at -20°C after fluorescence measurements and transported to the Smithsonian Marine Station in Fort Pierce, FL for further analyses.

Chlorophyll *a*, chlorophyll *b*, and carotenoid pigments were extracted from algae using standard methods and equations [54,55]. In brief, pigments were extracted from 2 subsamples of each algal individual in N,N-Dimethylformamide (DMF). Subsamples were homogenized in the DMF solvent with a handheld electric homogenizer and left to extract at 4°C for 24 hrs in total darkness. The extract was then centrifuged, and the supernatant was analyzed with a Genesys 180-UV Visible Spectrophotometer (Thermo Fisher Scientific, Waltham, MA) with 0.5 nm resolution. Pigment concentrations were normalized to subsample blotted mass and solvent volume.

### Macroalgal DOC production

Dissolved organic carbon (DOC) is released throughout the day as a function of photosynthetic activity. Considering this, we measured DOC fluxes by incubating algae within the experimental tanks for 4 hours during midday (1000–1400 EST). DOC flux measurements were taken at the beginning and end of the 25-day exposure period to measure the effect of pH treatment on DOC production. The tanks were cleaned before the incubation started. Several control tanks were added to the raceways to account for DOC flux in the water column. Samples were collected using pre-leached plastic 50 mL syringes with an attached filter holder containing a pre-combusted (450°C >4 hrs) 0.7 μM GF/F filter. Samples were stored in pre-combusted (550°C >4 hrs) 50 mL glass vials with Teflon-lined lids. Samples were immediately acidified to pH 2 to remove dissolved inorganic carbon and stored in the dark at 4°C. Samples were then shipped to Florida State University for subsequent analysis.

Replicate measurements of DOC were taken by high-temperature combustion using a Shimadzu TOC-L_CPH_ Total Organic Carbon Analyzer (Shimadzu, Kyoto, Japan) using the non-purgeable organic carbon (NPOC) methodology. The DOC concentration of each sample was calculated with standard methodology using a six-point standard curve as the mean of three to five injections using established protocols with a coefficient of variance less than 2% [56,57]. Deep seawater Consensus Reference Materials (CRMs) were analyzed to assess the precision and accuracy of the DOC analyses and deviated by < 5% from the reported values. DOC production by macroalgae was calculated as the change in DOC flux between the beginning and end of the exposure period and standardized by macroalgal biomass.

### Statistical analyses

Statistical analyses were performed using RStudio v1.2.5033 [58]. Data were checked for assumptions of independence, normality, and homogeneity of variances. When data did not meet these assumptions, they were square root transformed (Table 2) to achieve normal distributions. All data met the assumption of equal variances. One-way analyses of variances (ANOVA) were used to test the effect of seawater pH treatment on each response variable by species. All data are reported as mean ± 1 sd.

**Table 2:** Statistical analyses results.

Response Variable	Ambient pH (mean ± 1 sd)	Low pH (mean ± 1 sd)	F	p	Data Transformation
***Dictyota***					
Wet Weight (g)	0.434 ± 0.206	0.483 ± 0.389	0.016	0.901	Square Root
Net Photosynthesis (μmol g^-1^ h^-1^)	87.52 ± 65.71	90.33 ± 65.51	0.001	0.970	Square Root
Respiration (μmol g^-1^ h^-1^)	-21.69 ± 13.23	-22.39 ± 11.79	0.000	0.997	Square Root
Fv/Fm	0.610 ± 0.031	0.606 ± 0.024	0.068	0.798	Square Root
Chl. *a* (μg g^-1^)	148.702 ± 33.112	133.032 ± 19.326	1.503	0.238	
**Chl. *b* (μg g** ^**-1**^ **)**	**18.490 ± 4.899**	**13.875 ± 2.96**	**5.844**	**0.028**	
Total Carotenoids (μg g^-1^)	49.302 ± 11.081	44.899 ± 6.071	1.040	0.323	
***Halimeda***					
Buoyant Weight (g)	1.022 ± 0.308	0.954 ± 0.507	0.648	0.433	Square Root
Surface Area (cm^2^)	21.84 ± 8.33	20.34 ± 5.86	0.203	0.659	
Net Calcification (g d^-1^)	0.009 ± 0.004	0.002 ± 0.010	2.850	0.112	
Light Calcification (μmol g^-1^ h^-1^)	6.444 ± 9.115	8.248 ± 15.372	0.077	0.786	
Dark Calcification (μmol g^-1^ h^-1^)	-0.003 ± 0.004	-0.004 ± 0.003	0.926	0.351	
Net Photosynthesis (μmol g^-1^ h^-1^)	12.83 ± 6.47	11.59 ± 3.59	0.259	0.618	
Respiration (μmol g^-1^ h^-1^)	-6.78 ± 2.49	-7.19 ± 2.07	0.140	0.714	
Fv/Fm	0.487 ± 0.034	0.497 ± 0.043	0.319	0.581	
Chl. *a* (μg g^-1^)	92.559 ± 20.010	99.610 ± 16.901	0.617	0.444	
Chl. *b* (μg g^-1^)	73.036 ± 17.567	78.250 ± 11.737	0.543	0.472	
Total Carotenoids (μg g^-1^)	27.361 ± 6.189	29.242 ± 3.844	0.603	0.450	

The final column indicates when data transformation was needed to meet the assumptions (i.e., normality) of an ANOVA test.

## Results

### Macroalgal growth, calcification, net photosynthesis, and respiration

**Fig 1:**
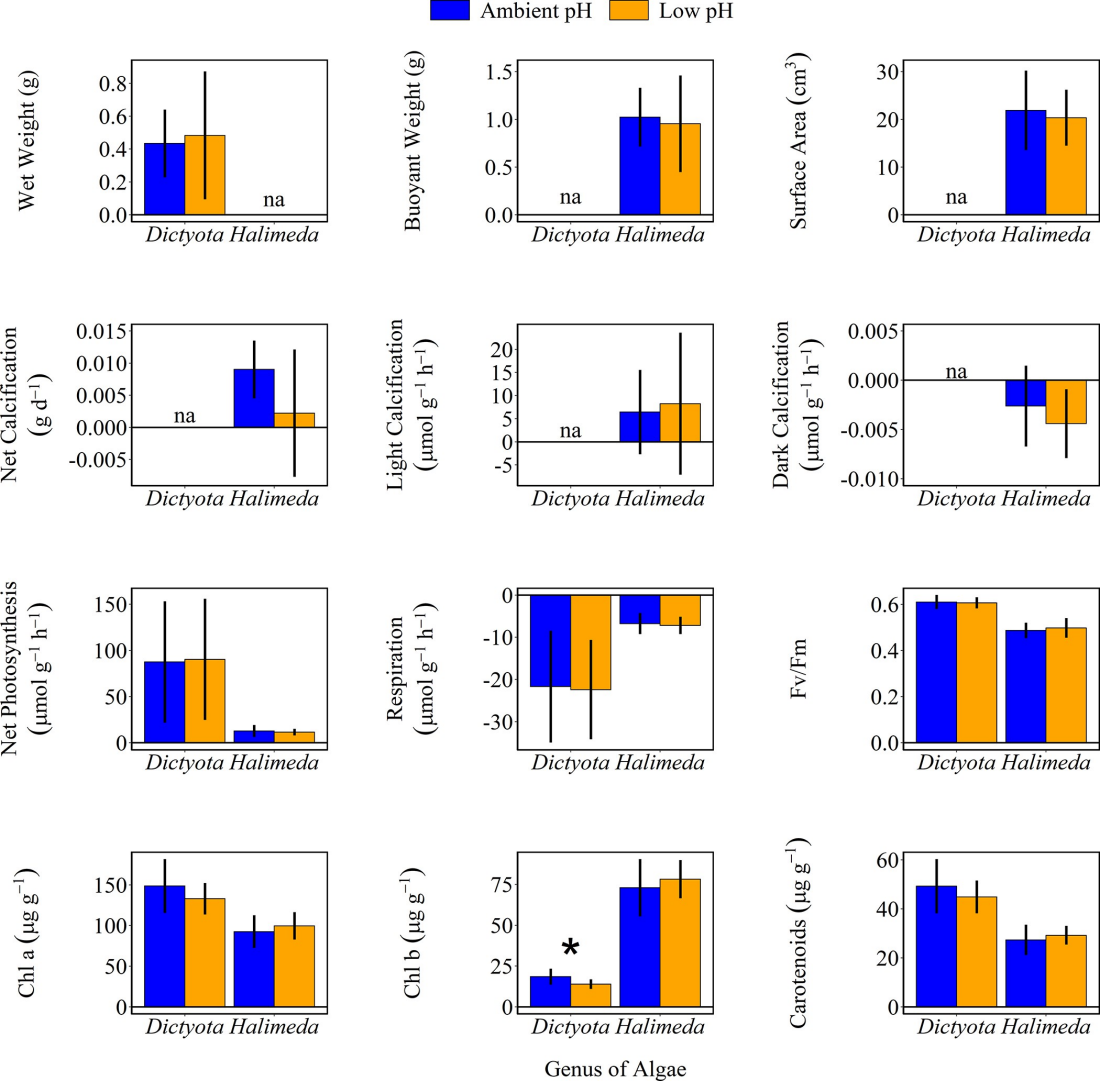
Photophysiology of *Dictyota* and *Halimeda* after 25 days of exposure to ambient pH (blue) and low pH (orange) conditions. Data are mean ± 1 sd. Asterisk indicates statistically significant differences (p< 0.05) between ambient and low pH treatments.

There were no significant differences in final wet weight (*Dictyota*), buoyant weight (*Halimeda*), or surface area (*Halimeda*) of either species between ambient and low pH treatments (Table 2 and Fig 1). For *Halimeda*, there were no significant differences in net, light, or dark calcification between ambient and low pH treatments. Net photosynthesis and respiration rates were consistent across pH treatments within species, although rates were different between species, with *Dictyota*’s rates being much higher than *Halimeda*’s.

### Macroalgal photophysiology and photosynthetic pigments

There were no significant differences in Fv/Fm, chlorophyll *a* concentration, or total carotenoids between ambient and low pH treatments in either species. However, there was a statistically significant difference in chlorophyll *b* concentration in *Dictyota* (F_1,X_ = 5.844, p < 0.03). *Dictyota* reared in low pH had lower levels of chlorophyll *b* compared to *Dictyota* reared in ambient pH (13.875 ± 2.96 μg g^-1^ versus 18.490 ± 4.899 μg g^-1^).

### Macroalgal DOC production

We did not detect a flux in the DOC produced by either species of macroalgae in the ambient or low pH treatment. With few exceptions, DOC fluxes were near zero and similar across control and algal incubations (S1 Fig). The minute differences between initial and final DOC measurements are most likely due to the seawater volume to algal biomass ratio being too high.

## Discussion

Both macroalgae species generally showed no changes in their growth or photophysiology after response to short-term exposure to acidification. This contrasts with our prediction that fleshy macroalgae (*Dictyota* spp.) would respond positively to elevated seawater *p*CO_2_, while calcifying macroalgae (*H*. *tuna*) would respond negatively. We were unable to quantify DOC flux due to limitations in the experimental design. To date, there have been only two studies that quantified the effects of short-term acidification on DOC fluxes by reef macroalgae. After 16 days of exposure to elevated seawater *p*CO_2_, *Halimeda opuntia* had higher daytime DOC production while *H*. *macroloba* had slightly lower daytime DOC production. There was no change in nighttime DOC fluxes for either of these calcifying macroalgae [59]. A more recent study showed non-calcifying macroalgae from the Great Barrier Reef had mixed responses to acidification with some algae having increased DOC release rates in light and dark after 1 and 19 days of exposure to elevated seawater *p*CO_2_ conditions [60].

The lack of response to low pH measured in this study is probably due to a combination of plasticity in carbon uptake mechanisms and environmental history experienced by these individuals. Brown and green macroalgae can use passive diffusion of CO_2_ from the water column and/or actively uptake bicarbonate ions (HCO_3_^-^) using carbon concentrating mechanisms (CCMs) [61–63]. While CCMs are an energetically costly form of carbon acquisition, it allows algae to use the most abundant inorganic carbon species in seawater [64] for photosynthesis. Some macroalgae can preferentially downregulate CCM activity when seawater CO_2_ concentrations are higher [30,32,65–67], thus saving energy which can be used for other physiological processes. The response of macroalgae to acidification also depends on its affinity for DIC (i.e., whether they are carbon-limited) and the environmental conditions that influence CCM function, such as light and nutrients [30,32,62,63,68–72].

It is not clear whether the species of *Dictyota* and *Halimeda* used in this study have the ability to downregulate CCM activity or if they are carbon limited. However, macroalgae with CCMs present, high affinity for DIC, and no change in CCM activity would not be expected to benefit from ocean acidification [66]. We detected no changes in growth of *Dictyota* or *Halimeda*. Therefore, we hypothesize they may possess the ability to regulate their CCM activity and show high affinity for DIC in seawater.

The responses of the specimens used in this study to elevated seawater *p*CO_2_ also may have been influenced by the natural variability in seawater carbonate chemistry and pH in the environment they were sourced from. Though we did not sample the carbonate chemistry of the site

from which these individuals were collected, inshore sites in the Florida Keys demonstrate wide seasonal fluctuations in seawater carbonate chemistry [73,74]. The lack of significant responses in these species of macroalgae, therefore, may reflect preadaptation to this variability in seawater pCO_2_. Overall, 47% of marine organisms originating from variable habitats have higher tolerance to ocean acidification [75]. However, the effects of environmental history on the tolerance of reef taxa to acidification are still unclear [76]. A few studies have shown contrasting effects of environmental history on the responses of crustose coralline algae (CCA) to acidification, demonstrating both increased [77,78] and decreased [77,79,80] stress tolerance. Thus, we can only speculate whether the species used in this study are more tolerant to acidification due to the natural fluctuations in *p*CO_2_ and pH experienced within the Florida Reef Tract.

Although there have been numerous studies examining the response of fleshy macroalgae to ocean acidification, *Dictyota* represents an under-studied genus [70]. There have been eight studies, including the present study, examining the physiological responses of *Dictyota* species to artificial acidification in laboratory experiments and across natural seawater pH and *p*CO_2_ gradients (Table 3). Short-term laboratory experiments lasting 1–4 weeks generally reported no statistically significant change in growth (Table 3), while field observations across pH gradients show increased benthic cover and abundance of *Dictyota* (and other non-calcifying algae) in regions with lower seawater pH [66,81–83]. This discrepancy may be due to several factors or, most likely, a combination of these factors. *Dictyota* may have increased reproduction and recruitment at sites with lower pH, either due to direct effects of increased *p*CO_2_ on algal physiology or indirect effects on outcomes of competitive interactions. Additionally, the increased benthic cover in the field represents generational effects with possible acclimation over generations, as demonstrated in coralline algae [84,85], thus leading to higher algal abundance closer to CO_2_ vents. Additionally, the magnitude of acidification may be responsible for the differences between studies; of the eight studies listed in Table 3, only one laboratory experiment [86] reported increased biomass of *Dictyota* under acidification, and this study used a *p*CO_2_ offset on the higher end of the ranges used. Field studies report similar or higher *p*CO_2_ gradients than those used in laboratory experiments. Macroalgae growing near CO_2_ vents also have likely experienced acidification for longer periods of time, possibly leading to different effects on growth (but see [87]). Field studies also include natural variations in other properties (i.e., light, temperature, and nutrients) that may enhance macroalgae growth in isolation or when combined with high seawater *p*CO_2_, so it is possible that the effects of these natural variations are not captured in laboratory experiments.

**Table 3:** *Dictyota* responses to elevated pCO_2_/lower pH in laboratory and field experiments. Interactive effects were not included in this literature review (i.e., responses to acidification only are recorded).

Citation	Species	General Location	Type of Study	Duration	pH and pCO_2_ Levels	Growth	Net Photo.	Resp.	Fv/Fm	Chl *a*	Chl *b*	Carotenoids	DOC Flux
Asnaghi et al. 2013 [88]	*D*. *dichotoma*	Bay of Villefranche, France	Lab	1 m	pH_T_: 8.09, 7.98, 7.84, 7.70 pCO_2_ (μatm): 382, 528, 755, 1093	−							
Burnham et al. 2022 [89]	*Dictyota* spp.	FRT	Lab	14 d	pH_T_: 7.90, 7.56 pCO_2_ (μatm): 581, 1420	↑	↓						
Ho and Carpenter 2017 [90]	*D*. *bartayresiana*	French Polynesia	Lab	7 d	pH: 8.04, 7.68 pCO_2_ (μatm): 404, 1065	−							
Johnson et al. 2014 [31]	*D*. *bartayresiana*	Palmyra Atoll	Lab	17 d	pH_SW_: 8.12, 7.99 pCO_2_ (μatm): 296, 431	−							
Page et al. 2021 [91]	*Dictyota* spp.	FRT	Lab	28 d	pH_T_: 7.86, 7.63 pCO_2_ (μatm): 603, 1105	−							
Porzio et al. 2020 [83]	*D*. *dichotoma* var. *intricata*	Ischia, Italy	Field	21 d	pH_SW_: 8.12, 7.99 pCO_2_ (μatm): 296, 431				−	−		−	
Rodriguez et al. 2018 [92]	*D*. *dichotoma*		Lab	17 d	pH_T_: 8.10, 7.60 pCO_2_ (μatm): 413, 1349	↓							
This Study	*Dictyota* spp.	FRT	Lab	25 d	pH_T_: 7.92, 7.67 pCO_2_ (μatm): 546, 1098	−	−	−	−	−	↓	−	?
					***Increase***	1	0	0	0	0	0	0	0
					***No Change***	5	1	1	2	2	0	2	0
					***Decrease***	1	1	0	0	0	1	0	0

The physiological responses of *Halimeda*, in contrast, have been well-studied since this macroalgae is an important contributor to calcium carbonate cycling and primary production in reef ecosystems. There have been 24 studies to date, including the present study, examining the biological responses of *Halimeda* species to seawater pH in experimental and natural settings (Table 4). Most studies reported the impacts of lower seawater pH on metabolic processes (e.g., calcification, photosynthesis, and respiration), with fewer studies documenting changes in photosynthetic pigments and efficiencies. With the exception of calcification rates, there were mostly no reported changes in the physiological responses of *Halimeda* species to elevated *p*CO_2_ and low pH (Table 4), as is shown in this study. Net calcification is the most well-documented response among *Halimeda* species with 60% of studies showing maintained rates under acidification and 40% of studies showing lower net calcification rates or net dissolution. Few species, including *H*. *heteromorpha*, *H*. *macroloba*, and *H*. *tuna*, appear to tolerate ocean acidification while other species like *H*. *cylindricea* are more sensitive to changes in carbonate chemistry. More research is needed to determine the physiological and environmental drivers of net calcification across species, including closely related ones.

**Table 4:** *Halimeda* responses to elevated pCO_2_/lower pH in laboratory and field experiments. Interactive effects were not included in this literature review (i.e., responses to acidification only are recorded).

Citation	Species	General Location	Type of Study	Duration	pH and pCO2 Levels	Net Calc.	Light Calc.	Dark Calc.	Net Photo.	Resp.	Fv/Fm	Chl *a*	Chl *b*	Carotenoids	Thallus Shedding	Bleaching	DOC Flux
Brown et al. 2019 [93]	*H*. *heteromorpha*	GBR	Lab	39 d	pH_SW_: 8.08, 7.80 pCO_2_ (μatm): 388, 631	−			−	−							
	*H*. *heteromorpha*	GBR	Lab	55 d	pH_SW_: 8.11, 7.82 pCO_2_ (μatm): 396, 631	−			−	−							
Campbell et al. 2014 [94]	*H*. *opuntia*	FRT	Lab	58 d	pH_T_: 8.08, 7.83, 7.68, 7.53, 7.33 pCO_2_ (μatm): 318, 611, 915, 1549, 2379	↓											
	*H*. *incrassata*	FRT	Lab	49 d	pH_T_: 7.95, 7.63, 7.32 pCO_2_ (μatm): 430, 1023, 2430	−											
	*H*. *simulans*	FRT	Lab	49 d	pH_T_: 7.95, 7.63, 7.32 pCO_2_ (μatm): 430, 1023, 2430	↓											
Campbell et al. 2016 [95]	*H*. *opuntia*	FRT	Lab	28 d	pH_NBS_: 8.01, 7.63 pCO_2_ (μatm): 491, 1402	↓			−	−							
	*H*. *incrassata*	FRT	Lab	28 d	pH_NBS_: 8.01, 7.63 pCO_2_ (μatm): 491, 1402				−	−							
	*H*. *simulans*	FRT	Lab	28 d	pH_NBS_: 8.01, 7.63 pCO_2_ (μatm): 491, 1402	−			−	−							
Comeau et al. 2013 [96]	*H*. *macroloba*	French Polynesia	Lab	14 d	pH_T_: 8.19, 8.04, 7.94, 7.81, 7.70, 7.41 pCO_2_ (Pa): 26, 40, 53, 75, 102, 211	−											
	*H*. *minima*	French Polynesia	Lab	14 d	pH_T_: 8.19, 8.04, 7.94, 7.81, 7.70, 7.41 pCO_2_ (Pa): 26, 40, 53, 75, 102, 211	↓											
Comeau et al. 2014 [97]	*H*. *macroloba*	French Polynesia	Lab	14 d	pH_T_: 8.05, 7.83, 7.71 pCO_2_ (μatm): 391, 726, 993	−											
	*H*. *macroloba*	Hawaii	Lab	10 d	pH_T_: 8.01, 7.83, 7.71 pCO_2_ (μatm): 430, 696, 959	−											
	*H*. *macroloba*	Okinawa	Lab	7 d	pH_T_: 7.99, 7.84, 7.73 pCO_2_ (μatm): 449, 679, 921	−											
Hofmann et al. 2014 [35]	*H*. *opuntia*	Caribbean	Lab	28 d	pH_NBS_: 8.126, 7.736 pCO_2_ (μatm): 415, 1705	−					−						
Hofmann et al. 2015 [98]	*H*. *opuntia*	Caribbean	Lab	28 d	pH_T_: 8.03, 7.77 pCO_2_ (μatm): 447, 872	↓			−	−							
Johnson et al. 2014 [31]	*H*. *taenicola*	Palmyra Atoll	Lab	9 d	pH_SW_: 7.56, 7.45 pCO_2_ (μatm): 1330, 1365	−											
	*H*. *taenicola*	Palmyra Atoll	Lab	15 d	pH_SW_: 8.00, 7.29 pCO_2_ (μatm): 400, 2314	−											
	*H*. *opuntia*	Palmyra Atoll	Lab	17 d	pH_SW_: 7.22, 7.02 pCO_2_ (μatm): 1116, 2178	↓											
McNicholl et al. 2019 [99]	*H*. *scabra*	FRT	Lab	4 h	pH_T_: 8.05, 7.68 pCO_2_ (μatm): 417, 1190		−		−								
McNicholl et al. 2020 [36]	*H*. *scabra*	FRT	Lab	13 h	pH_T_: 7.98, 7.63 pCO_2_ (μatm): 506, 1354			↓		−							
McNicholl & Koch 2021 [100]	*H*. *scabra*	FRT	Lab	5 d	pH: 8.07, 7.71 pCO_2_ (μatm): 367, 992	−	↑	↓	−	−							
	*H*. *copiosa*	Caribbean	Lab	5 d	pH: 8.07, 7.71 pCO_2_ (μatm): 367, 992	−	−	−	−	−							
	*H*. *goreauii*	Caribbean	Lab	5 d	pH: 8.07, 7.71 pCO_2_ (μatm): 367, 992	↓	↓	−	−	−							
Meyer et al. 2015 [101]	*H*. *macroloba*	GBR	Lab	16 d	pH_T_: 8.038, 7.707 pCO_2_ (μatm): 402, 996	−	−	−	−	−	−	−					−
	*H*. *opuntia*	GBR	Lab	16 d	pH_T_: 8.038, 7.707 pCO_2_ (μatm): 402, 996	−	−	↓	−	−	−	−					−
**Citation**	**Species**	**General Location**	**Type of Study**	**Duration**	**pH and pCO2 Levels**	**Net Calc.**	**Light Calc.**	**Dark Calc.**	**Net Photo.**	**Resp.**	**Fv/Fm**	**Chl *a***	**Chl *b***	**Carotenoids**	**Thallus Shedding**	**Bleaching**	**DOC Flux**
Meyer et al. 2016 [102]	*H*. *incrassata*	Caribbean	Lab	10 d	pH_NBS_: 8.22, 7.84 pCO_2_ (μatm): 377, 1076		↓			−	−						
Peach et al. 2016 [103]	*H*. *discoidea*	FRT	Lab	27 d	pH_NBS_: 8.06, 8.00, 7.86, 7.74 (NBS) pCO_2_ (μatm): 491, 653, 982, 1201	−											
Peach et al. 2017 [104]	*H*. *incrassata*	Caribbean	Lab	42 d	pH_NBS_: 8.20, 8.02 pCO_2_ (μatm): 487, 789	−									−		
	*H*. *tuna*	Caribbean	Lab	42 d	pH_NBS_: 8.20, 8.02 pCO_2_ (μatm): 487, 789	−									−		
	*H*. *monile*	Caribbean	Lab	42 d	pH_NBS_: 8.20, 8.02 pCO_2_ (μatm): 487, 789	−									−		
	*H*. *opuntia*	Caribbean	Lab	42 d	pH_NBS_: 8.20, 8.02 pCO_2_ (μatm): 487, 789	−									−		
	*H*. *copiosa*	Caribbean	Lab	42 d	pH_NBS_: 8.20, 8.02 pCO_2_ (μatm): 487, 789	−									−		
	*H*. *goreauii*	Caribbean	Lab	42 d	pH_NBS_: 8.20, 8.02 pCO_2_ (μatm): 487, 789	↓									−		
Price et al. 2011 [105]	*H*. *opuntia*	Palmyra Atoll	Lab	14 d	pH_SW_: 8.0, 7.7 pCO_2_ (μatm): 440, 946	↓									↑		
	*H*. *taenicola*	Palmyra Atoll	Lab	14 d	pH_SW_: 8.0, 7.7 pCO_2_ (μatm): 440, 946	↓									−		
Ries et al. 2009 [106]	*H*. *incrassata*	Cape Cod, MA	Lab	60 d	pH levels not reported pCO_2_ (μatm): 409, 606, 903, 2856	↓											
Scherner et al. 2016 [107]	*H*. *cuneata*	Brazil	Lab	23 d	pH: 8.10, 7.85, 7.60, 7.20 pCO_2_ (ppm): 400, 560, 1140						−						
Sinutok et al. 2011 [108]	*H*. *macroloba*	GBR	Lab	28 d	pH_NBS_: 8.1, 7.9, 7.7, 7.4 pCO_2_ (Pa): 32, 67, 108, 247	−					↓	−	−				
	*H*. *cylindracea*	GBR	Lab	28 d	pH_NBS_: 8.1, 7.9, 7.7, 7.4 pCO_2_ (Pa): 32, 67, 108, 247	↓					↓	↓	↓				
Sinutok et al. 2012 [109]	*H*. *macroloba*	GBR	Lab	28 d	pH_NBS_: 8.1, 7.7 pCO_2_ (μatm): 381, 1208	↓					↓	↓	↓				
	*H*. *cylindracea*	GBR	Lab	28 d	pH_NBS_: 8.1, 7.7 pCO_2_ (μatm): 381, 1208	↓					↓	↓	↓				
Vogel et al. 2015a [110]	*H*. *digitata*	Papua New Guinea	Field	14 d	pH_T_: 8.14, 7.83 pCO_2_ (Pa): 33, 86		↑	−	−	↑							
	*H*. *opuntia*	Papua New Guinea	Field	14 d	pH_T_: 8.14, 7.83 pCO_2_ (Pa): 33, 86		↑	↓	−	−							
Vogel et al. 2015b [111]	*H*. *opuntia*	GBR	Lab	16 d	pH_T_: 8.038, 7.707 pCO_2_ (μatm): 421, 1069	−	−	↓	−	−		−					
Wei et al. 2020 [112]	*H*. *cylindracea*	China Sea	Lab	28 d	pH_NBS_: 7.62, 7.80, 8.11 pCO_2_ (ppm): 400, 1000, 1600	↓					↓	↓		−			
	*H*. *lacunalis*	China Sea	Lab	28 d	pH_NBS_: 7.62, 7.80, 8.11 pCO_2_ (ppm): 400, 1000, 1600	↓					↓	↓		−			
This Study	*H*. *tuna*	FRT	Lab	25 d	pH_T_: 7.92, 7.67 pCO_2_ (μatm): 546, 1098	−	−	−	−	−	−	−	−	−	−	−	?
					***Increase***	0	3	0	0	1	0	0	0	0	1	0	0
					***No Change***	24	6	5	16	16	6	5	2	3	8	1	2
					***Decrease***	16	2	5	0	0	6	5	3	0	0	0	0

## Conclusion

Ocean acidification will impact coral reef ecosystems through direct effects on organismal physiology and indirect effects on ecosystem processes. Biological responses of fleshy and calcifying macroalgae can modify reef biogeochemistry, potentially leading to impaired coral health and altered food web cycling via sponge and microbial loops [23]. In this study, we found that ocean acidification does not impact the photophysiology of two reef macroalgae found on the Florida Reef Tract, *Dictyota* and *Halimeda*. Our results build on previous literature that documents a lack of significant changes in biological responses to ocean acidification in these genera (Tables 3 and 4) and other benthic calcifying macroalgae [37]. However, the species used in this study, while common on coral reefs across the globe reefs, are under-studied. Therefore, additional research should be conducted to determine genera- and species-specific responses to ocean acidification across varying regions and environmental conditions to determine the physiological and environmental mechanisms driving growth and primary production in reef macroalgae. This will create a more informed pathway towards studying and modeling subsequent indirect effects of changing seawater biogeochemistry on coral reef ecosystem processes, including DOC cycling through microbial and sponge loops, coral-algal competition, and algal community structure on reefs.

## Supporting information

S1 Fig DOC fluxes. DOC fluxes for control and algal (*Dictyota* and *Halimeda*) incubations under ambient (blue) and low pH (orange) at the beginning (left) and end (right) of the 25-day exposure period.
(TIF)
