# Supplementary figures and images for: Effects of ocean acidification on growth and photophysiology of two tropical reef macroalgae
Source: PLoS One. 2023 Nov 17;18(11):e0286661. doi: 10.1371/journal.pone.0286661 (PMC10655979; doi:10.1371/journal.pone.0286661)

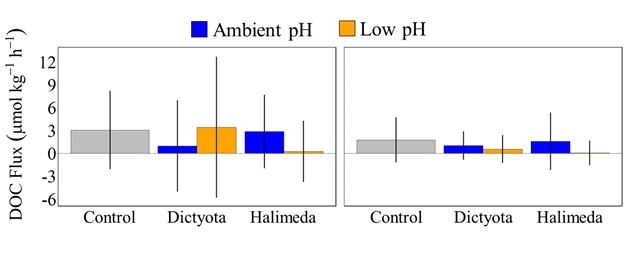

Supplement: S1 Fig — DOC fluxes for control and algal (Dictyota and Halimeda) incubations under ambient (blue) and low pH (orange) at the beginning (left) and end (right) of the 25-day exposure period. (TIF) [file pone.0286661.s001.tif]
